# Brightness Invariant Deep Spectral Super-Resolution

**DOI:** 10.3390/s20205789

**Published:** 2020-10-13

**Authors:** Tarek Stiebel, Dorit Merhof

**Affiliations:** Institute of Imaging & Computer Vision, RWTH Aachen University, 52072 Aachen, Germany; Dorit.Merhof@lfb.rwth-aachen.de

**Keywords:** spectral super-resolution, spectral signal recovery, spectral reconstruction from RGB

## Abstract

Spectral reconstruction from RGB or spectral super-resolution (SSR) offers a cheap alternative to otherwise costly and more complex spectral imaging devices. In recent years, deep learning based methods consistently achieved the best reconstruction quality in terms of spectral error metrics. However, there are important properties that are not maintained by deep neural networks. This work is primarily dedicated to scale invariance, also known as brightness invariance or exposure invariance. When RGB signals only differ in their absolute scale, they should lead to identical spectral reconstructions apart from the scaling factor. Scale invariance is an essential property that signal processing must guarantee for a wide range of practical applications. At the moment, scale invariance can only be achieved by relying on a diverse database during network training that covers all possibly occurring signal intensities. In contrast, we propose and evaluate a fundamental approach for deep learning based SSR that holds the property of scale invariance by design and is independent of the training data. The approach is independent of concrete network architectures and instead focuses on reevaluating what neural networks should actually predict. The key insight is that signal magnitudes are irrelevant for acquiring spectral reconstructions from camera signals and are only useful for a potential signal denoising.

## 1. Introduction

Spectral imaging itself offers benefits for a variety of applications throughout computer vision. All applications that can profit from spectral imaging belong on an abstract level to one of the areas of color science or remote sensing. The terms spectral, multi-spectral and hyper-spectral are broadly utilized and are not well defined. In fact, there exist multiple and distinct definitions for the same terms depending on the current field, e.g., remote sensing or colorimetry. The major commonality is that spectral imaging is a generic term for both multi-spectral and hyper-spectral imaging. Hyper-spectral imaging makes it possible to measure the continuous spectrum. In contrast, multi-spectral imaging only allows to sample the spectrum at a higher resolution, since it utilizes more channels than RGB devices have. The greatest ambiguity lies in the view of what is a sufficient representation of a continuous spectrum.

Although spectral imaging techniques were researched for decades and multiple, distinct systems were proposed, there is always a tradeoff between the spatial resolution, temporal resolution, spectral resolution and costs. On the most abstract level, all spectral imaging systems (HSI) can be subdivided into two classes: scanning and snapshot. Scanning techniques perform a true hyper-spectral image acquisition by either scanning the spatial domain, e.g., push-broom, or the spectral domain, e.g., filter-wheel/liquid crystal tunable (LCTF) based cameras. The major limitation of such systems is that they are limited to static scenes.

Snapshot spectral imaging explicitly aims at achieving a high temporal resolution. This usually leads to a reduced spectral and/or spatial resolution. They should therefore not be considered as hyper-spectral imaging systems, but rather as multi-spectral systems, since the precise spectral signature must first be computed from not ideally sampled data points. Snapshot spectral imaging is thus closely related to computational spectral imaging. Computational based spectral imaging gained particular interest in recent years due to the continuously growing capacity of technology that fosters modern, learning based algorithms, especially deep learning. Computed tomography imaging spectrometers (CTIS) [1,2,3] employ dedicated gratings to disperse spectral stimuli in multiple directions. As a result, multiple dispersed images are captured by the imaging sensor, where each dispersed image might be viewed as a two-dimensional projection of the three-dimensional spectral cube. Although CTIS are in theory capable of a high temporal resolution, the post processing effort is immense. Coded aperture snapshot imaging (CASSI) [4,5] can be viewed as a further development of CTIS. CASSI is based on compressive sensing and offers an interesting solution but fails yet to achieve a great reconstruction quality. In analogy to modern RGB imaging devices, there is the comparably new development of multi-spectral color filter arrays (MSCFA). Although they were already considered for a long time theoretically [6], their manufacturing is not trivial, leading to effects such as a varying spectral sensitivity across the sensor or viewing angle instabilities. Also, there are drawbacks regarding the spatial resolution.

Spectral reconstruction from RGB or spectral super-resolution (SSR) is a computational spectral imaging approach which, in contrast to dedicated spectral imaging systems, only requires cheap camera technology in the form of conventional end-consumer devices. It therefore promises a cheap spectral imaging solution for mass market adoption. The recovery of spectral signatures from RGB signals is a heavily underconstrained problem that can hardly be solved by classical signal processing techniques. Known SSR approaches almost exclusively rely on machine-learning with the underlying premise that the computer may find patterns that are beyond human understanding. To this end, machine learning requires large datasets for training. In the context of SSR, such a dataset ideally consists of corresponding pairs of RGB images and spectral images. Real world image pairs (RGB and spectral) are usually not available. However, there exist large datasets of spectral images only. It is current practice to simulate the corresponding RGB images from the spectral data. Such generated image pairs lay the foundation for not only training modern, data driven approaches, but also for evaluating them.

Pioneering work on SSR utilized radial basis function networks [7] and sparse coding [8,9,10]. Today, they are outperformed by modern convolutional networks, which have continuously demonstrated superior results. The recent NTIRE2020 challenge on spectral reconstruction from RGB [11] as well as its predecessor in 2018 [12] provide a concise overview over the state-of-the-art methods. In summary, the best performing methods in terms of spectral reconstruction quality exclusively consist of complex convolutional neural networks [13,14,15,16,17,18,19]. A major limitation of deep learning based approaches for SSR is their susceptibility to a varying brightness, i.e., signal scale. It was first noted by Lin et al. [20], who investigated the potential scale invariance of the leading entries of the NTIRE 2018 Challenge on spectral reconstruction. Scale invariance was basically found to be non-existent. An additional analysis for scale invariance was conducted at the 2020 challenge on spectral reconstruction from RGB [11]. The leading methods were shown to once again be susceptible to changes in brightness, although their general robustness increased. However, the improved robustness resulted from a superior, more general database that implicitly included varying scales.

Changes in signal scale occur frequently in practical applications. The most intuitive example is a varying exposure time. A probably more critical application relying on brightness invariance is video surveillance, in particular the case of moving objects under a fixed light source. Concrete examples might be video based patient monitoring in a clinical setting or car driver monitoring. If the monitored person moves such that the relative position to the camera/light source changes, the spectral stimuli incident on the sensing device will change in scale (neglecting effects such as specular reflections). Such movements should obviously not cause inherently different spectral reconstructions, potentially leading to false alerts.

Up to the authors knowledge, there exist no published articles on achieving brightness invariance for deep learning based SSR. Yet, utilizing deep learning for SSR is desirable since it forms the state-of-the-art in terms of spectral reconstruction quality. So far, the only known way to approach the issue is from a data side by either employing data augmentation [20] or directly utilizing more complex databases [11]. If it is possible to ensure that the training data is diverse and covers all possibly occurring signal intensities, brightness invariance can be learned by the network from the data itself. However, obtaining spectral databases of such diversity requires an immense effort and is often not feasible. When spectra occur whose intensities are lower or higher than what is known from the training data, the signal processing will become unstable.

Within this work, we aim to provide a general solution for deep learning methods that guarantees scale invariance for SSR at all times independent of the training data. The contributions of this work are:

- We investigated a modern sparse coding technique to highlight why it is scale invariant by construction: only the signal vector directions are of relevance, not the signal magnitude!
- We transferred the gained insights to reformulate the prediction goal for deep learning based SSR.
- As a result, we propose a fundamental deep learning based approach for SSR that is invariant to scale.

## 2. Method

### 2.1. Image Formation

The process of image formation is commonly modeled assuming a Lambertian world,

(1) $$\varphi_{i}=\int_{\lambda_{min}}^{\lambda_{max}}r\left(\lambda \right)i\left(\lambda \right)s_{i}\left(\lambda \right)d\lambda ,$$

with r(λ) denoting the spectral object reflectance, i(λ) the spectral power distribution of the illuminant and $s_{i}\left(\lambda \right)$ the response function of the $i^{\prime }th$ camera channel. The above equation is for convenience, mostly considered in a discretized version

(2) φ=Sr,

with the matrix S $\in R^{3xq}$ denoting the spectral weights, i.e., the combination of the camera response and an illuminant, uniformly sampled at a total of *q* wavelengths. Within this work, we consider the wavelengths ranging from 400nm to 700 in 10 nm steps, corresponding to q=31 spectral dimensions.

Modern image processing algorithms in form of convolutional neural networks do not process only individual signals but entire images. Spectral images are denoted as $I^{s}\left(x,y\right)$ and camera images as $I^{c}\left(x,y\right)$, where *x* and *y* are the pixel positions. The pixel positions are discarded, unless they are explicitly required. RGB images, which is denoted by $I_{ideal}^{c}$, can be created by applying Equation (2) on every pixel position of a spectral image. However, such an idealized simulation is hardly realistic. An obvious example might be the color filter array used in most real world RGB imaging devices: only one of the three channels is actually measured at every pixel position. In order to simulate more realistic RGB images, the ideal images are subjected to further processing steps. The additional processing steps of the ideal camera image $I_{ideal}^{c}$ will be denoted by P,

(3) $$I_{out}^{c}=P\left(I_{ideal}^{c}\right)$$

In analogy to the well known challenges on spectral reconstruction from RGB [11,12], distinct processing pipelines, called scenarios, are considered within this work. The different scenarios are:

- **Ideal**
  The ideal images were used without any further modification, i.e., P becomes an identity mapping.
- **Clean**
  The ideal images were subjected to quantization, assuming they are 8-bit.
- **Real**
  The ideal images were subjected to a sequence of processing steps to emulate realistic behavior. They consist in the order of: subsampling (assuming an RGGB Bayer filter array), adding shot noise (Poisson distribution) and dark noise (normal distribution), quantization (8 bit) and demosaicing.

Assuming a known pair of a spectral image, $I_{gt}^{s}$, and an RGB image, $I^{c}$, we can define spectral signal recovery as any functional, R, that allows for the estimation of the spectral image from the camera image

(4) $$\begin{array}{c} I_{rec}^{s}=R\left(I^{c}\right). \end{array}$$

The estimated spectral image should minimize a chosen error metric,

(5) $$\min err(I_{rec}^{s},I_{gt}^{s}).$$

The spectral recovery is invariant to scale, if

(6) $$\alpha I_{rec}^{s}=R\left(\alpha I^{c}\right)$$

for any arbitrary scalar α∈R. It is easy to verify that deep convolutional neural networks do **not** have this property.

### 2.2. Sparse Coding for SSR

The breakthrough for sparse coding based spectral reconstruction can most likely be attributed to the work of Arad [8] in 2016, although there exist comparable research [9,10]. The general idea behind the approach is to learn an overcomplete dictionary of spectral signals from a spectral prior/dataset utilizing a clustering analysis, e.g., K-SVD [21] which is a generalization of the k-means clustering method. The resulting dictionary, can be seen as a single matrix with the columns corresponding to the spectral signals, so-called atoms. The dictionary is subsequently projected onto the camera signal space given a known camera response, leading to an associated dictionary of camera signals,

(7) $$D_{hs}=\left\{{\vec{h}}_{1},{\vec{h}}_{2},\ldots ,{\vec{h}}_{n}\right\},$$

(8) $$D_{rgb}=\left\{{\vec{\varphi }}_{1},{\vec{\varphi }}_{2},\ldots ,{\vec{\varphi }}_{n}\right\}.$$

In the original work [8], a dictionary of the size of n=500 was employed. Any RGB camera signal $\vec{\varphi }$ can be spectrally super-resolved with the help of the dictionary by finding a sparse linear combination of atoms, e.g., utilizing orthogonal matching pursuit (OMP) [22],

(9) $$\vec{\varphi }=D_{rgb}\vec{w},$$

where the weight vector $\vec{w}$ is subject to sparsity constraints. The weight vector uniquely defines the associated spectral reconstruction

(10) $${\vec{r}}_{rec}=D_{hs}\vec{w}.$$

**Figure 1:**
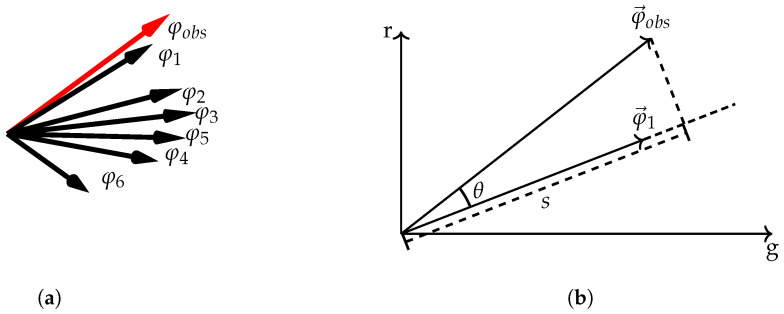
Visualization on how dictionary based spectral reconstruction propagates the signal scale to the spectral domain. In the simplest case, the associated atom for an observed camera signal is the nearest neighbor of the dictionary (**a**). The signal scale is extracted by vector projection (**b**).

It should be noted that such a sparse coding approach is scale invariant by design. The key insight is that the dictionary based method is only dependent on the direction of signal vectors, but not their magnitude. This can be explained by the process of finding the proper atoms for an observed camera signal, which is qualitatively visualized in Figure 1a. All the black arrows denote atoms within the RGB camera space, i.e., the dictionary. For simplicity, we only consider the borderline case of having a sparsity constraint of one. This means that given an observed camera signal, the nearest neighbor in terms of the angular error would be chosen as the corresponding atom, which in term is directly linked to a spectral signal. The observed RGB signal, $\varphi_{obs}$, is shown in red. The nearest atom is $\varphi_{1}$. A vector projection of the nearest atom onto the observed camera signal directly yields the signal scale. Differences in scale can be propagated to the spectral domain due to the linearity involved in obtaining the RGB atoms by linear projection. This approach of scale extraction by vector projection is visualized in Figure 1b.

### 2.3. Deep Scale Invariance

Scale invariance can be acquired by shifting from considering absolute signals, both RGB and spectral, to only vector directions. This might seem counter intuitive at first glance, especially in the context of data analysis, since possible information in form of the vector magnitudes is actively neglected. However, we claim that the discarded information is completely irrelevant for spectral signal recovery.

We begin to outline the steps necessary to make common network architectures for SSR scale invariant. As the very first processing step, every RGB signal, ${\vec{\varphi }}_{in}$, at every pixel position of the input RGB image is independently normalized to have unit length, ${\vec{\varphi }}_{n}=\frac{{\vec{\varphi }}_{in}}{|{\vec{\varphi }}_{in}|}$. We call this step *scale normalization*. It can be seen as a different form of instance normalization in the context of deep learning.

**Figure 2:**
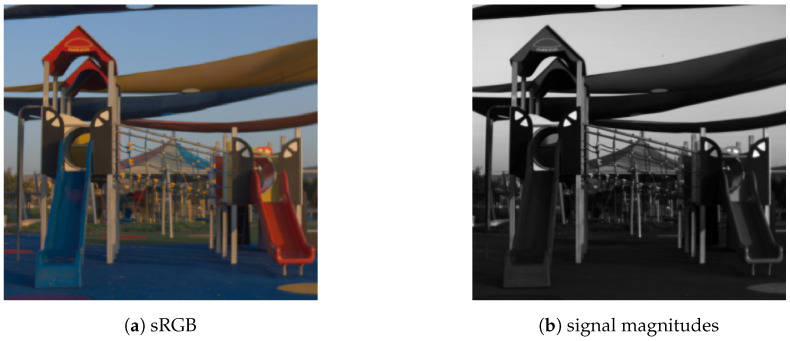
An exemplary image of the spectral dataset [11] once rendered in sRGB assuming CIE D50 lighting in the original scene (**a**) as well as the corresponding signal magnitudes per pixel (**b**).

The Euclidean length of all input signals could be stored for all pixel positions resulting in a luminance type of image as shown in Figure 2. However, only the normalized camera signals are forwarded. All the signal magnitudes are completely ignored. It is important to stress that by following this approach, signal information is actively neglected. The normalized RGB image is processed by a convolutional neural network (CNN) to obtain a spectral reconstruction. Any CNN might be employed. Developing a new network architecture is not the focus of this work, but how to achieve brightness invariance independent of the concrete network. Therefore, different CNNs from the current state-of-the-art will be considered later on. The network output is insensitive to the input signal scale since any potential scale was removed by the initial normalization. Although it is not strictly necessary, we again normalize every spectral signal as reconstructed by the neural network to unit length, since it is computationally more robust. The learning objective is thus shifted from learning a direct end-to-end mapping from RGB-signals towards spectral signals to only considering the vector directions. When considering sparse coding based techniques, our proposed prediction task might be viewed as learning a mapping from RGB signals towards an atom of the dictionary.

The missing scale in the spectral domain is estimated in analogy to dictionary based approaches. Any spectrum predicted by the neural network, ${\vec{r}}_{n}$, is back-projected into camera RGB space utilizing the known camera response and Equation (2), resulting in ${\vec{\varphi }}_{rec}$. Subsequently, it is necessary to minimize the distance from an arbitrarily scaled ${\vec{\varphi }}_{rec}$ to the input RGB signal,

(11) $$s_{spec}=\underset{s}{\arg\;\min}(|s{\vec{\varphi }}_{rec}-{\vec{\varphi }}_{in}\left|\right).$$

The scale is thus obtained from the vector projection of ${\vec{\varphi }}_{in}$ on ${\vec{\varphi }}_{rec}$

(12) $$s_{spec}=\frac{\langle {\vec{\varphi }}_{rec},{\vec{\varphi }}_{in}\rangle }{|{\vec{\varphi }}_{rec}|},$$

where 〈,〉 denotes the inner product.

The spectral prediction is finally computed as the scaled version of the normalized network output,

(13) $${\vec{r}}_{rec}=s_{spec}{\vec{r}}_{n}.$$

All of the steps described for scale propagation can in fact be implemented in form of fixed layers in a deep neural network. The combination of these layers are, in the following sections, called *scale propagation*. Based on the original input camera signal, the spectral estimate is scaled appropriately.

**Figure 3:**
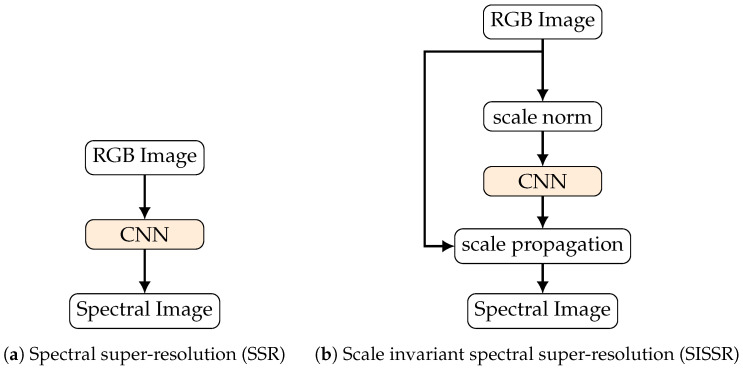
Visualization of the proposed workﬂow for achieving scale invariance using deep learning based spectral spectral reconstruction methods.

The complete workflow for our proposed scale invariant spectral super-resolution (SISSR) is depicted in Figure 3. It is a generic approach and completely independent of the network architecture utilized.

## 3. Experiments

**Figure 4:**
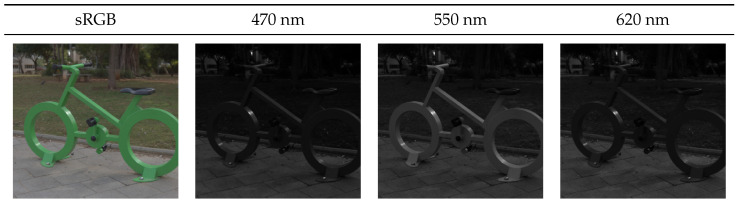
An exemplary image from the dataset NTIRE 2020 [11].

The NTIRE2020 [11] dataset was utilized for evaluation, as it forms the newest and largest spectral dataset to date. It consists of a 450 spectral images ranging from 400 to 700 nm in 10 nm steps and with a spatial resolution of 512×482 pixels. All images were captured outdoors in Israel, mostly at bright daylight. Exemplary images of the dataset are shown in Figure 2a and Figure 4. For better human interpretability, the spectral images were rendered in sRGB assuming CIE D50 illumination. The dataset was subdivided into three splits: training, validation and testing. Our test set equals the official validation split of the challenge to allow for an easy comparability. Corresponding RGB images were respectively computed from all spectral images for the three scenarios ideal, clean and real utilizing the CIE 1964 human standard observer. It should be noted that the original challenge was subdivided into the two tracks “Clean” and “RealWorld”. The “Clean” track equals our scenario “quantization”. The “RealWorld” track is similar to our scenario “real”. However, the camera sensitivity was not disclosed within the challenge track. Since knowing the camera sensitivity is essential for our workflow, we thus computed our own “real” images as described in the scenario by assuming a known camera response function (CIE 1964 standard observer).

We adopted the most common error metrics to report results. The *mean relative absolute error* (MRAE) established itself not only as one of the standard evaluation metrics for the spectral reconstruction quality, but also as the go-to loss function for SSR,

(14) $$MRAE\left(I_{rec}^{s},I_{gt}^{s}\right)=\frac{100}{MNq}\sum_{x=1}^{N}\sum_{y=1}^{M}\sum_{\lambda =\lambda_{min}}^{\lambda_{max}}\frac{|I_{rec}^{s}\left(x,y,\lambda \right)-I_{gt}^{s}\left(x,y,\lambda \right)|}{I_{gt}^{s}\left(x,y,\lambda \right)}.$$

where *M* and *N* respectively denote the image width and height. In contrast to most other published research, we report the MRAE in percentile, because we believe that it offers a better readability. Thus, there is the scaling factor of 100. Additionally, the root mean squared error (RMSE), and the spectral angle mapper (SAM) in degree, are considered.

(15) $$RMSE\left(I_{rec}^{s},I_{gt}^{s}\right)=\sqrt{\frac{1}{MNq}\sum_{x=1}^{N}\sum_{y=1}^{M}\sum_{\lambda =\lambda_{min}}^{\lambda_{max}}{\left(I_{rec}^{s}\left(x,y,\lambda \right)-I_{gt}^{s}\left(x,y,\lambda \right)\right)}^{2}},$$

(16) $$SAM\left(I_{rec}^{s},I_{gt}^{s}\right)=\frac{1}{MNq}\sum_{x=1}^{N}\sum_{y=1}^{M}angle\left(I_{rec}^{s}\left(x,y\right),I_{gt}^{s}\left(x,y\right)\right),$$

### 3.1. Networks and Training Details

In order to evaluate the proposed methodology, distinct network architectures from the current state-of-the-art methods are considered: the HSCNN+R [17], an adopted UNet [18], the adaptive weight attention network (AWAN) [15] and the pixel-aware deep function-mixture network (FMNet) [13]. The respective code is publicly available for all individual network architectures. Since all implementations are available in Python and based upon the deep learning framework Pytorch, the proposed workflow was likewise implemented in Python/Pytorch and allows us to exchange the different network architectures on a modular basis. In following such an approach, it is possible to rely on a single, unified workflow for training and evaluation. All methods but the modified UNet [18] rely on ensemble strategies to further push their performance, mostly self-ensemble and model-ensemble.

Although ensemble methods allow us to optimize a model’s performance further, it does not add anything to the focus of this work while significantly increasing the computational overhead. We therefore perform the experiments without ensemble methods.

There are multiple ways to configure the different network architectures, in particular due to the ensemble strategies applied by different authors. Since we do not consider ensemble strategies but instead focus on a singular network configuration, the precise configurations for every neural network as well as further relevant implementation details are summarized:

- **UNet**
  There only exists a single configuration that was proposed in [18] and no model ensemble methods were employed.
- **HSCNN-R**
  This network architecture is a residual network that was optimized for SSR. The main network configuration as proposed in [17] having 64 filters in each layer and a total of 16 resblocks were utilized. The weights were initialized according to the algorithm proposed by He et al. [23].
- **AWAN**
  We considered the fully stacked configuration consisting of the basic AWAN network combined with the patch-level second-order non-local (PSNL) and the adaptive weighted channel attention (AWCA) modules as reported in [15]. The amount of dual residual attention blocks (DRAB) is set to 8 with 200 output channels. The AWCA module has a reduction ratio of t=16 and the PSNL model an *r*-value of 8.
- **FMNet**
  The utilized configuration [13] consists of two FM blocks with each block containing three basis functions. Each basis function as well as the mixing functions are formed by two convolutional blocks having 64 feature maps. The initial learning rate is halved every 20 epochs and the training ends after 100 epochs.

Finally, Table 1 provides an overview of additional hyperparameters per method that are required for training.

Using these settings, each network is trained in each scenario from scratch in both its original form (SSR) as well as within our proposed scale invariant SSR (SISSR) approach.

The training details therefore remain identical for both SSR and SISSR training and do not need any modification.

### 3.2. Results and Discussion

Table 2 offers a first concise overview of the reconstruction results. When only considering all methods in their original form, they perform as would be expected from known benchmarks. The reconstruction results are generally best within the ideal scenario and worst within the real scenario. This is due to the increased amount of disturbances introduced in the RGB image creation. Of particular interest for this work is the performance of all neural networks when applied within our scale-invariant approach. The influence of our methodology can be summarized as a significant performance boost within the ideal scenario, while showing a detrimental effect in terms of average reconstruction error within both the clean and real scenario. The larger the disturbances within the RGB images, the bigger is the performance gap between SSR and SISSR. This result is consistent for all network architectures.

An intuitive explanation for the observed results can be found. If the input RGB image is subjected to disturbances, any deficits in the RGB signals will obviously lead to a deterioration of the spectral reconstruction. This is under the assumption that the signal processing is unable to fully compensate for poor image quality, i.e., the neural networks implicitly learn to denoise RGB images. However, our proposed scale propagation additionally introduces a way for the input RGB image to bypass the neural network in order to correctly propagate any signal scale. In following such an approach, not only the signal scale, but also errors within the RGB images will be directly propagated to the spectral domain. This conclusion can even be visualized.

**Figure 5:**
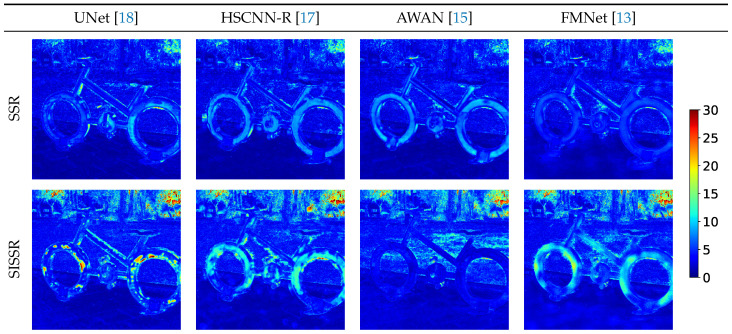
Qualitative visualization of the achieved reconstruction quality. For each method, the prediction error (MRAE) is shown per pixel.

Figure 5 displays the spectral reconstruction errors per pixel in terms of MRAE for an exemplary image from our test set in the clean scenario. The RGB images within this scenario are only disturbed by quantization noise. Thus, the disturbances within the RGB signals are the more severe the darker the image regions become. The spectral reconstruction can therefore be expected to suffer for dark pixels, especially when using our SISSR approach. This is directly visible in Figure 5, for example in the top right corner of all images. All models already have trouble accounting for the high noise levels in their original form. Within the SISSR approach, the image noise is additionally propagated to the spectral domain leading to even worse reconstruction results. For noiseless image regions, the SISSR approach has a varying effect on the spectral reconstruction quality. For some regions, the results are better, for others, the results are worse. On average, the spectral reconstruction quality increases for well illuminated image regions. This can be best validated by the fact that a significant performance increase is consistently achieved in the ideal scenario for all networks when used in our SISSR approach.

**Figure 6:**
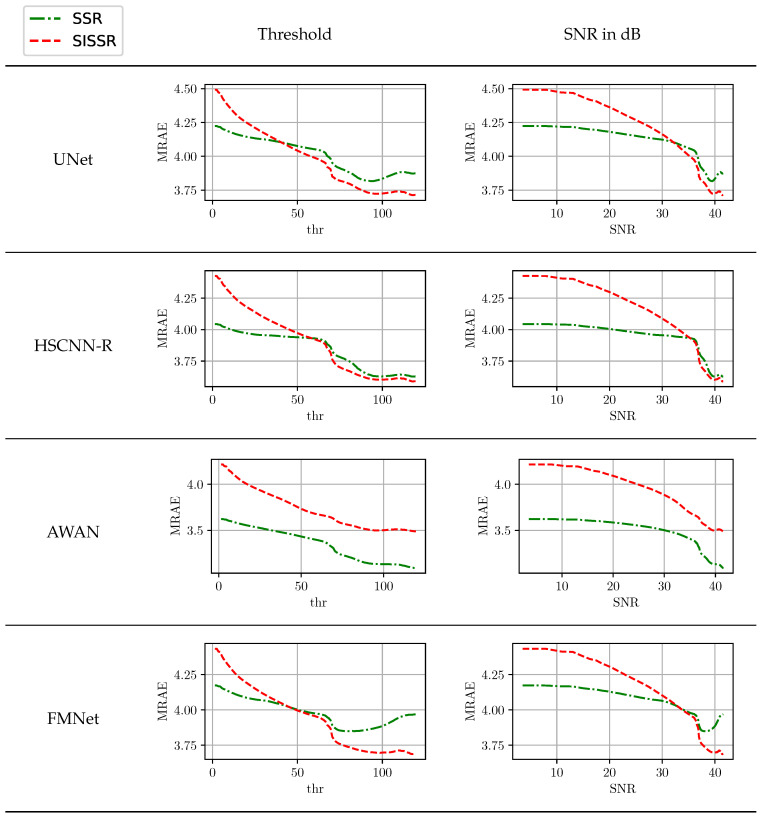
The inﬂuence of RGB signal noise on the spectral reconstruction quality.

From the discussed results, the question arises as to how different noise levels in the RGB image effect the spectral reconstruction. For this purpose, we carried out an adapted evaluation in which only pixel positions where the RGB signal magnitude is greater than a threshold value contribute towards the average spectral reconstruction errors. Figure 6 displays the achieved reconstruction results for all methods in the clean track over the threshold value. Additionally, the chosen threshold values can directly be converted into associated signal–noise ratios (SNR). The worst SNR associated with a threshold value can be approximated by the mean quantization error for the bit just above the threshold. For example, if the threshold value would be 12, the worst quantization errors are introduced within the signal range [12, 13] which are all clipped to 12. By assuming the simplification that all ideal RGB signal magnitudes are uniformly distributed, threshold values are converted to an associated SNR. Due to the involved simplifications in computing the SNR, the provided results are not 100% accurate, but they are more than sufficient to get a general idea of the influence of SNR on the reconstruction quality. The higher the SNR, the better the reconstruction results. However, all neural networks in their original form are somewhat robust against low SNR values and consistently yield a comparable performance. Only minor benefits are gained from better signals. In contrast, the proposed method shows a stronger dependence on low noise levels. Its performance consistently increases with an increasing SNR until it surpasses the original workflow. For all network architectures but AWAN, the break-even point for SSR and SISSR is at an SNR of approximately 35 dB. The AWAN network is in comparison to the other considered architectures by far the most complex, as it comprises the largest amount of trainable parameters.

It is again stressed that the proposed SISSR approach completely removes any scale on the input data before it is processed by the neural network which therefore has less information available. Considering the pure task of spectral reconstruction neglecting additional, implicit functionality of neural networks such as denoising, there is no decrease in performance, indicating that camera signal scale is irrelevant for spectral signal recovery. In fact, there was a major performance gain observed in the ideal scenario, which can be attributed to a more restricted solution space through our proposed approach. However, noisy RGB images have a stronger effect on the SISSR approach due to its limited denoising capabilities.

### 3.3. Brightness Invariance and Ablation Study

**Figure 7:**
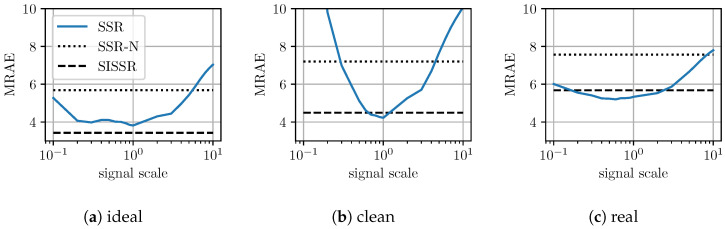
Detailed visualization of the results reported in Table 2 and Table 3 for the UNet architecture.

**Figure 8:**
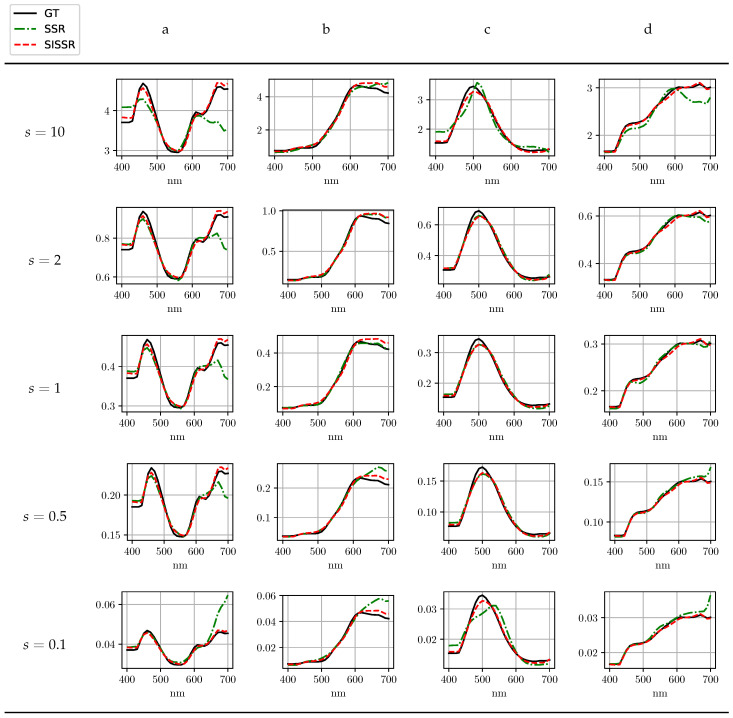
Exemplary reconstruction results for the adaptive weight attention network (AWAN) network [15] under varying scales. The pixel positions a–d are shown in Figure 9.

The most important advantage of the proposed approach is its inherent robustness with respect to changes in image brightness. In order to evaluate the robustness of all methods regarding changes in image brightness, all spectral images within the test set as well as the corresponding RGB images were scaled by a scalar, *s*. Utilizing the scaled RGB-images, all previously trained networks were again tasked with spectral signal recovery. The average reconstruction results for SSR are shown in Table 3 at different scales. In contrast, the different scales do not effect the proposed workflow (SISSR). The results that were already reported for SISSR in Table 2 are therefore independent of any changes in image brightness and may serve as comparison. This is due to the explicit normalization conducted within the SISSR workflow that completely removes different brightness levels. For a convenient comparison of all the results, Table 3 is printed next to Table 2. When considering a halving, s=0.5, or doubling, s=2, the spectra recovered by SSR get worse on average, but for the majority of spectra, the results are still reasonable in terms of their general shape. Assuming strong differences in scale, s=0.1 or s=10, the spectral reconstructions become unstable and thus unreliable. Only the results achieved for SSR at scale values of s=0.5 and s=2 in the real scenario can be interpreted as about equal to SISSR in terms of averaged metrics over the test images. When comparing SSR and SISSR at different scale values, SISSR outperforms SSR in most cases. For a better intuition on the results reported in Table 3, Figure 7 exemplary visualizes the achieved results for the UNet architecture in the three distinct scenarios for both SSR and the proposed SISSR approach. The average spectral reconstruction error is plotted over different scaling factors in logarithmic scale. The dotted and constant line represents the proposed SISSR approach. In contrast, SSR appears in the shape of a parabola and is therefore limited to signal scales close to one. Finally, Figure 8 shows distinct reconstructed spectra for the AWAN network which overall performed the best at different scales. It can be observed that in particular for strong changes in image brightness, the shape of the recovered spectra collapses for SSR whereas SISSR remains robust.

The proposed workflow for scale invariance (SISSR) consists of two steps:

(1) Training a CNN to only predict the shape of the spectra up to scale from the RGB input.
(2) Adjusting the brightness (scale) of the recovered spectra to match the input in post-processing, i.e., Equations (12) and (13).

The question may arise if both steps are indeed necessary for achieving scale invariance since step two can be applied independently of step one. Therefore, an ablation study was considered. All spectra as they were recovered by the standalone neural networks (SSR) are subsequently subjected to the second processing step of brightness adjustment, i.e., they are post-processed in the same way as within SISSR. This approach is referred to as SSR-N. The major difference between SISSR and SSR-N is that for SISSR the CNNs were trained to predict the shape of the spectra up to an arbitrary scale due to the explicit normalization layer, whereas for SSR-N, the CNNs were trained to predict the very precise spectra due to no normalization layer. SSR-N could therefore be interpreted as a baseline SISSR has to outperform. The results for SSR-N are shown in Table 2 to allow for a direct comparison to previous results. Indeed, SSR-N is consistently inferior to SISSR in terms of spectral reconstruction quality yielding the conclusion that only considering the post-processing in form of step two is insufficient. However, SSR-N is in contrast to SSR robust to changes in image brightness as it can be observed in Figure 7. This demonstrates the explicit need to normalize the input RGB to enforce a shape of the recovered spectra that only depends on the direction of the RGB input vectors. When there is no normalization, the CNN may in the worst case recover two distinct spectra in terms of shape when observing separate highly saturated colors that only differ in brightness such as a dark red and a bright red. Such behavior is not reasonable from a physical perspective. Subsequently adjusting the scale of the ill-shaped spectra to better approximate to observed signal in RGB space is not beneficial.

Finally, the argument can be made that the RGB normalization layer might not be necessary when a scale invariant loss function is considered. Examples might be the spectral angular error, spectral information divergence [24] or spectral derivative based loss functions [25]. It should be noted that from a purely theoretical point of view there is no guarantee that training a network with a loss function that is invariant to changes in brightness will make the fully-trained network invariant to changes in brightness. However, the exploration of these loss functions is beyond the scope of this manuscript and might be interesting to investigate in future research.

## 4. Conclusions

A new method was proposed for deep learning based spectral super-resolution which, in contrast to before, allows any deep learning based spectral reconstruction algorithm to gain the important property of brightness invariance/signal scale invariance. The proposed approach is based on the assumption that only the directions of both RGB and spectral signals are of relevance for the recovery of spectral signals, not the actual signal magnitude. This enables a better generalization and offers spectral reconstructions that are not only more reliable, but also better understandable by humans for practical applications. Analogue to sparse coding based techniques, signal scale propagation is achieved by backprojection into camera signal space. Consequently, emphasis is implicitly placed on predicting the general shape of the spectra. The proposed approach does not effect the inference time since the additional processing steps are computationally extremely light weight in comparison to a modern CNN architecture.

It was demonstrated that a significant performance gain can be observed when considering ideal signals, suggesting that the proposed approach limits the solution space of neural networks in a physically meaningful way. However, it was found that the proposed approach has a higher susceptibility to noise than utilizing neural networks alone, trained in classical end-to-end fashion. Although the proposed approach remains stable under the presence of noise, the averaged reconstruction quality in terms of metrics such as the mean relative absolute error is worse. An analysis was provided on how different SNR values of the input RGB image affect the spectral reconstruction quality. The break even point for neural networks as a stand-alone and in conjunction with our approach is approximately at an SNR of 35 dB. For higher noise levels, neural networks as a stand-alone perform better. For lower noise levels, the proposed approach provides superior performance.

The most significant advantage of the proposed approach is its complete robustness regarding changes in image brightness. While all network architectures alone fail to robustly offer a spectral reconstructions under varying brightness levels, such differences do not effect the proposed workflow. The proposed workflow even outperforms the stand-alone networks at higher noise levels as soon as the image brightness/exposure differs compared to the training data. It should be noted that our brightness invariant approach has the most value when the training set is small and does not cover all possible signal intensities that might occur in practice since it achieves a better generalization. Should the training data be diverse and all signal intensities of the test set fall within the ranges that are known from the training set, brightness invariance can be learned by a network from the data itself.

## Figures and Tables

**Figure 9:**
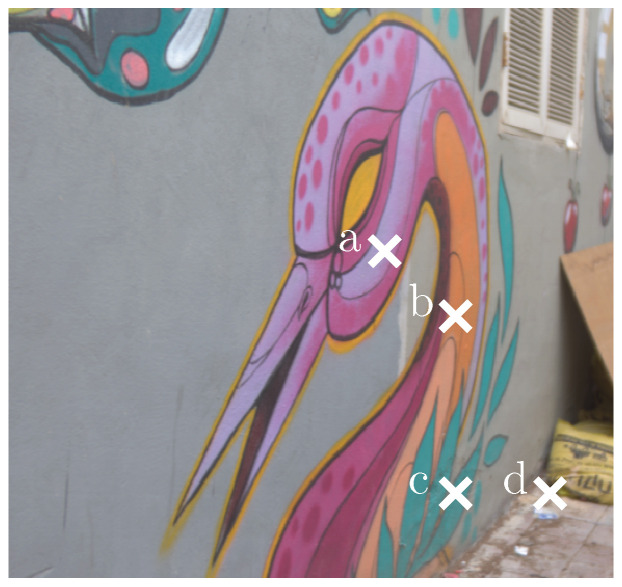
An exemplary image from the test set (in sRGB) which is associated with the reconstruction results shown in Figure 8.

**Table 1:** Utilized hyperparameters for training the different networks.

	Batch Size	Patch Size	Learning Rate	Optimizer	Loss
UNet [18]	10	32	1 × 10^−3^	Adam	MRAE
HSCNN-R [17]	64	50	2 × 10^−4^	Adam	MRAE
AWAN [15]	16	64	1 × 10^−4^	Adam	MRAE
FMNet [13]	128	64	1 × 10^−4^	Adam	L1

**Table 2:** Average reconstruction metrics over the test set, which equals the official validation split of the NTIRE 2020 challenge on spectral reconstruction [11]. Spectral angle mapper (SAM) is reported in degree, mean relative absolute error (MRAE) in percent.

		UNet [18]			HSCNN-R [17]			AWAN [15]			FMNet [13]		
		MRAE	RMSE	SAM	MRAE	RMSE	SAM	MRAE	RMSE	SAM	MRAE	RMSE	SAM
**ideal**	SSR	3.817	0.016	2.670	3.601	0.014	2.487	3.276	0.013	2.322	3.799	0.016	2.686
	SSR-N	5.684	0.021	3.964	6.619	0.023	4.135	4.742	0.016	3.325	5.392	0.021	3.849
	SISSR	3.430	0.014	2.480	3.420	0.015	2.485	2.999	0.011	2.132	3.363	0.014	2.453
**clean**	SSR	4.224	0.016	2.850	4.044	0.015	2.733	3.655	0.013	2.444	4.173	0.016	2.783
	SSR-N	7.203	0.026	4.626	7.102	0.023	4.351	6.454	0.021	3.869	6.353	0.021	3.891
	SISSR	4.492	0.016	2.869	4.426	0.016	2.949	4.214	0.014	2.778	4.433	0.016	2.893
**real**	SSR	5.333	0.016	3.003	5.175	0.016	2.985	4.821	0.015	2.802	5.111	0.015	2.976
	SSR-N	7.569	0.022	4.279	7.573	0.021	4.103	7.147	0.021	4.043	7.978	0.025	4.377
	SISSR	5.678	0.016	2.981	5.483	0.016	3.085	5.698	0.017	3.290	5.657	0.017	3.211

**Table 3:** Average reconstruction metrics for varying scales assuming the original workflow—spectral super-resolution (SSR). SAM is reported in degree, MRAE in percent.

		UNet [18]				HSCNN-R [17]				AWAN [15]				FMNet [13]			
scale		0.1	0.5	2	10	0.1	0.5	2	10	0.1	0.5	2	10	0.1	0.5	2	10
**ideal**	MRAE	5.272	4.105	4.298	7.035	5.514	4.020	4.074	7.220	3.890	3.351	3.432	4.892	5.203	4.534	4.053	22.120
	RMSE	0.001	0.003	0.009	0.097	0.001	0.003	0.010	0.119	0.000	0.002	0.007	0.094	0.001	0.002	0.010	0.295
	SAM	3.403	2.255	1.918	5.378	5.187	2.725	1.894	6.002	1.823	1.865	1.798	5.087	4.023	2.220	1.983	6.295
**clean**	MRAE	20.440	5.112	5.264	10.085	23.010	4.951	4.922	8.138	4.838	3.974	4.283	7.164	21.259	4.925	4.944	24.761
	RMSE	0.002	0.003	0.010	0.098	0.003	0.003	0.015	0.101	0.001	0.003	0.010	0.091	0.003	0.003	0.010	0.210
	SAM	7.147	2.635	2.308	5.671	8.679	3.094	2.623	5.952	2.951	2.769	2.313	4.531	7.619	2.800	2.401	5.746
**real**	MRAE	6.013	5.234	5.530	7.800	6.807	5.137	5.535	6.968	5.084	4.485	4.700	6.595	7.195	5.205	5.361	30.158
	RMSE	0.001	0.004	0.010	0.075	0.001	0.003	0.010	0.084	0.001	0.003	0.009	0.104	0.001	0.004	0.010	0.303
	SAM	4.223	2.732	2.370	4.433	4.484	2.859	2.519	4.736	4.172	2.607	2.207	5.674	4.666	2.966	2.434	7.378
